# The Measurement of Adult Pathological Demand Avoidance Traits

**DOI:** 10.1007/s10803-018-3722-7

**Published:** 2018-08-23

**Authors:** Vincent Egan, Omer Linenberg, Elizabeth O’Nions

**Affiliations:** 1 Centre for Forensic and Family Psychology, University of Nottingham, Yang Fujia Building, Jubilee Campus, Wollaton Road, Nottingham, NG8 1BB UK; 2 Faculty of Psychology and Educational Sciences, Parenting and Special Education Research Unit, KU Leuven, Leuven, Belgium; 3 Leuven Autism Research (LAuRes), KU Leuven, Leuven, Belgium; 4 MRC Social, Genetic and Developmental Psychiatry Centre, Institute of Psychiatry, Psychology and Neuroscience, King’s College London, London, UK; 5 Division of Psychology and Language Sciences, Developmental Risk & Resilience Unit, Clinical, Educational, and Health Psychology Research Department, University College London, London, UK

**Keywords:** Extreme demand avoidance, Autistic spectrum disorder, Pathological demand avoidance, Personality, Offending, Asperger’s syndrome

## Abstract

Pathological (“extreme”) demand avoidance (PDA) involves obsessively avoiding routine demands and extreme emotional variability. It is clinically linked to autism spectrum disorder (ASD). The observer-rated EDA Questionnaire (EDA-Q) for children was adapted as an adult self-report (EDA-QA), and tested in relation to personality and the short-form Autism Screening Questionnaire (ASQ). Study 1 (n = 347) found the EDA-QA reliable, univariate, and correlated with negative affect, antagonism, disinhibition, psychoticism, and ASQ scores. Study 2 (n = 191) found low agreeableness, greater Emotional Instability, and higher scores on the full ASQ predicted EDA-QA. PDA can screened for using this tool, occurs in the general population, and is associated with extremes of personality. Future studies will examine if PDA occurs in other clinical populations.

**Electronic supplementary material:**

The online version of this article (10.1007/s10803-018-3722-7) contains supplementary material, which is available to authorized users.

Pathological demand avoidance (PDA) is a behavioural profile associated with apparently obsessive non-compliance, distress, and florid challenging and socially inappropriate behaviour in children, adolescents and adults (Newson et al. 2003; O’Nions et al. 2014b). PDA is associated with a passive early history over the first year of development; avoidance of demands, with extreme outbursts if demands are escalated; surface sociability but apparent lack of sense of social identity; lability of mood and impulsivity; comfort in role play and pretending; language delay, possibly attributable to passivity; obsessive behaviour; and soft neurological signs (awkwardness, clumsiness, dyspraxia and similar) (Newson et al. 2003). Some propose a terminological move from ‘pathological’ to ‘extreme’ demand avoidance. This is to reflect the idea that, from the individual’s perspective, avoidance of everyday requests may seem appropriate and thus not ‘pathological’, even though if disproportionate to others, hence the alternative term, EDA (Gillberg 2014). For others, the chronicity of the problem justifies the term “pathological”.

Broadening diagnostic criteria over the last 30 years means many of the 12 children Newson originally described would now likely meet diagnostic cut-offs for ASD. Indeed, one study found similar levels of autistic traits in children identified as having PDA compared to a sample with ASD not selected for PDA (O’Nions et al. 2014a). PDA traits appear to exist in varying concentrations within ASD (e.g. O’Nions et al. 2016; Gillberg et al. 2015). This is congruent with the broader literature on ASD sub-populations who exhibit severe non-compliance and emotional dysregulation (e.g. Lucyshyn et al. 2004). Behavioural non-compliance and emotional dysregulation is not exclusive to ASD, but have plausible drivers in the context of ASD [e.g. sensory sensitivities, phobias, need for predictability/sameness, perception of routine requests as aversive (Lucyshyn et al. 2004; O’Nions et al. 2018)].

Early work comparing ASD to PDA conceptualized PDA as a separate subgroup (Newson et al. 2003). This work observed that persons with PDA reject demands through a variety of social strategies, such as distraction or negotiation, whereas individuals with ASD tend to be more forthright and direct, so less strategic or ‘manipulative’ in their rejection of demands. Given the concept of autism has broadened to include a wider range of phenotypes, social methods of distraction are likely to be seen across a broader cross section of the autism spectrum. In addition, demand avoidance in individuals with PDA was reportedly unselective; enjoyable activities were as likely to be rejected as stressful ones. This suggests that demands in themselves were aversive for these individuals. Impulsivity and immediate mood changes were also reported more in individuals with PDA, whereas individuals with ASD were noted to lack impulsivity and adhered to routine. Recent work in individuals with ASD and problem behavior suggests a robust correlation between non-compliance with routine requests and irritability (Chowdhury et al. 2016). Also differentiating ASD and PDA, there is some evidence to show individuals with PDA show good imagination and role-playing [although reports of observed behaviour suggest this often involves mimicking characters and stories rather than introducing novelty (O’Nions et al. 2018)]. A similar degree of obsessionality may also be present—although with a more social focus in individuals with the PDA phenotype relative to those with more ‘typical’ ASD (Newson et al. 2003).

PDA is informally recognised by some practitioners and some service-user groups in the UK and beyond, but has remains controversial. While parents and carers observe and report associated behaviours, PDA is not currently included in diagnostic manuals, and research on the topic is in its infancy. There is debate as to whether the PDA profile represents a set of behaviours that can occur across many diagnostic profiles, a pattern of comorbidity of multiple behavioural syndromes, or is a distinct profile in itself, perhaps signifying future diagnoses (Vizard 2008; O’Nions et al. 2014a, b).

Irrespective of underlying drivers, there is little doubt individuals with PDA sometimes present with very problematic behavior, including aggression, socially maladaptive behaviours, and, commonly, educational placement breakdown (O’Nions et al. 2014a, 2016; Gore Langton and Frederickson 2016). Anecdotal reports suggest parents and teachers of persons with PDA-like behaviour struggle to manage unpredictable and volatile behaviour. While one study found that PDA in the context of ASD reduces from child to adulthood (Gillberg et al. 2015), another suggested that less than half of individuals experienced an improvement comparing reports of past to current severity of behaviours (O’Nions et al. 2016).

Research on PDA has not yet considered adult populations, partly because no reliable tool has been available for use in systematic studies of these features in adults. Moreover, work has predominantly focused on PDA traits in individuals with diagnosed ASD, with less attention given to individuals with a broader autism phenotype, which lies on a behavioural and genetic continuum with diagnosed autism (Constantino and Todd 2003, 2005; Robinson et al. 2016). Comorbidity of different mental disorders is common within ASD conditions (Doshi-Velez et al. 2014; Gillberg and Billstedt 2000).

The current research describes the adaptation of an informant-rating instrument (the Extreme Demand Avoidance Questionnaire; O’Nions et al. 2014b; EDA-Q) for use as a self-report measure of traits and behaviours related to PDA in adults without intellectual impairment (Extreme Demand Avoidance Questionnaire—Adult version; EDA-QA). This was done by rephrasing the items of the observer-rated EDA Questionnaire (EDA-Q) for children into equivalent propositions which an adult responded to on a 5-point likert scale, Study 1 validates this scale. In Study 1, we use this measure to examine the relationship between PDA traits, ASD traits, and other psychopathology dimensions, in a community sample of adults reporting self-identified psychopathology. The internal and external reliability and validity of the EDA-QA is examined by testing whether greater scores are associated with concurrent callous–unemotional behaviour, general traits associated with personality disorder, and ASD features. On the basis of previous work highlighting overlap between PDA, ASD, anxiety, and maladaptive behaviour (O’Nions et al. 2014a, b, 2016), we predicted that EDA-QA scores would share variance with several other psychopathology dimensions, particularly those associated with problem behavior and emotional dysregulation.

## ASD, PDA and Offending Behaviour

Forensic psychologists have become interested in the incidence of ASD and the broader autism phenotype in offender populations of the kind considered by Criminal Justice Systems (CJS; Trundle et al. 2017). While a systematic review of persons with ASD by King and Murphy (2014) did not find persons with ASD disproportionately over-represented in the CJS, they noted that some persons were more implicated in a variety of crimes, and that individuals with apparent psychiatric comorbidity had more CJS involvement. Im (2016) has argued that generative features (e.g., comorbid psychopathology, social-cognition deficits, and emotion-regulation problems) and associational issues (e.g., younger age, Asperger’s syndrome diagnosis, and repetitive behaviour) may increase risk of violence in this population.

The PDA phenotype may have forensic implications. In addition to oppositional behavior, a small follow-up study of adults identified as having PDA as children found they also had problems with mood, social vulnerability, violence, and stalking behaviour (Newson et al. 2003), some of which could increase risk of CJS involvement. As such, we seek to explore the links between PDA and offending behaviour, and the second study presented here examines the relationship between PDA traits and self-reported delinquency in the community, taking into account personality disposition, empathising, and ASD traits.. Specifically, the study examined the predictive relationship between PDA and offending behaviour, over and above these other factors.

The present sample differs from participant groups previously studied in the context of work on PDA. Specifically, we recruited a high-functioning general population sample self-reporting mental health problems interested in ASD and PDA, and assessed them for traits associated with these conditions. This is the first study to address PDA and ASD difficulties in a community sample of this kind. Given ASD and PDA traits were predominantly self-identified, we tested how much the self-diagnosis was congruent with actual behaviour and dispositional characteristics.

## Study 1

### Method and Procedure

#### Participants

Participants were recruited from a variety of specialist on-line blogs and community forums focusing on the needs and concerns of persons with ASD. In all cases, the host of the forum or blog was contacted and made aware of the nature of the study and its intention, and no study related materials or links were uploaded until the moderators of the website gave their approval. Sites hosting the link to the study were The PDA Society (UK) (http://www.pdasociety.org.uk); Special Needs Jungle (UK) (http://www.specialneedsjungle.com/); Aspies Central (USA; https://www.aspiescentral.com/); Julia Daunt (UK; http://www.memyselfandpda.com); two closed Facebook pages: Pathological Demand Avoidance UK, and ‘Aspergers is another part of the spectrum’; The Neurotypical Site (Australia) (http://www.theneurotypical.com/index.html), and Autistic Spectrum Australia (http://www.autismspectrum.org.au/). Data for the study were collected over the Internet using Bristol On-Line Surveys (http://www.survey.bris.ac.uk). Participants were invited to share the link throughout their own social network, allowing further crowdsourcing of the sample.

##### Description of the Cohort

The study recruited 347 persons via the above-mentioned forums/social networks [94 males, 230 females, 19 other (self-declared ‘non-binary/gender fluid’), 4 persons omitted gender information]. The mean age of participants was 36.9 years (SD = 12.8 years, range 18–84), with a mean of 14.7 years education (SD = 3.6 years), indicating a highly educated group. Of the sample, 21% reported that they had been previously arrested, and 11.8% had a prior conviction; all convictions were for minor offences. Over half of the cohort (54.4%) reported a formal prior mental health diagnosis: 97 (28%) depression; 58 (16.7%) an ASD diagnosis; 53 (15.3%) an anxiety disorder; 28 (8.1%) ADHD/ADD; 24 (6.9%) personality disorder, and 14 (4%) PTSD; two individuals claimed prior drug problems, and one reported gender dysphoria. There was therefore a significant incidence of concurrent mental disorders of various kinds. Of 332 who answered questions about mental disorder diagnosis, 62 (18.6%) had no concern they had mental health problems or an actual psychiatric diagnosis; 85 (26.6%) persons believed they had a mental disorder which had not been diagnosed; 41 (12.3%) believed they did not have a disorder despite receiving a psychiatric diagnosis; and 144 (43.3%) accepted a formally diagnosed mental disorder. Of the 129 self-evaluation reports for self-identified psychiatric or developmental conditions, 29 individuals reporting self-identified ASD also reported having PDA, 44 persons claimed to have PDA alone, and a further 19 self-identified PDA alongside depression or anxiety; separately, 59 persons claimed to have formally diagnosed ASD. While the cohort were well educated and predominantly female, they had a disproportionate level of mental-health difficulties, in that half of the group have had a diagnosed mental health problem at some point in their life, with a further sizeable proportion (over 26%) having concerns of an undiagnosed mental disorder.

#### Ethics

The study was prepared according to British Psychological Society guidelines for research with human participants and on-line research and passed by the University ethics committee and all external agencies involved. No person was under any compulsion to participate, and no individual was identifiable from their personal data.

#### Design

The current study sought to test the psychometric structure and internal validity of the EDA-QA, and the relationship between it’s score and measures of ASD, callous–unemotional traits and traits associated with personality disorder, including negative affect, detachment, antagonism, disinhibition, and ‘psychoticism’ (schizotypal indicators such as eccentricity, cognitive perceptual dysregulation, and unusual beliefs and experiences). We also examined the predictive relationship between demographic factors, reported clinical diagnoses and PDA. Assuming an effect size of 0.15, a p value of < .05, and a sought power of 0.95, with 18 (maximum) predictors, a linear regression model testing successive blocks changing the R^2^ would require at least 213 participants (Faul et al. 2009) A large cohort spanning a range of psychopathology traits was expected to provide substantial variability across the dimensions of interest. Some of these individuals were expected to self-identify as having traits associated with PDA.

#### Measures

The study used four self-report instruments (and one observer rating). All were relatively brief, minimising participant burden. These scales were:

##### The EDA-QA

The EDA-QA is a 26-item self-report adaptation of the observer-rated EDA-Q (O’Nions et al. 2014a) revised for use with adult populations. The original EDA-Q for children showed good sensitivity and specificity for the construct and had an internal reliability (Cronbach’s alpha) of 0.87. Exploratory item factor analysis suggested that the majority of EDA-Q items loaded onto a single factor. The instrument was used by parents rating their children who showed extreme and challenging behaviour. Four versions of the new self-report version were prepared: these asked about EDA behaviour as a child versus as an adult and were completed by the participant or their nominated rater. Conversion of the EDA-Q to the EDA-QA simply involved making items age-appropriate, and changing the tense of original EDA survey items to equivalent self-report propositions, to be scored on a 4 point rating scale (“not true”, “somewhat true”, “mostly true”, and “very true”, scored 1–4, respectively) (see Table 1 for adult items).

**Table 1:** Item factor analysis (oblique rotation, pattern matrix) of the EDA-QA (n = 347)

Item	Factor 1	Factor 2
11. I am good at getting round others and making them do as I want	**0.79**	− 0.17
25. I seek to quibble and change rules set by others	**0.78**	− 0.05
15. I have a very rapidly changing mood (e.g., I can switch from affectionate to angry in an instant)	**0.77**	0.05
3. I am driven by the need to be in charge	**0.77**	− 0.17
17. I blame or target a particular person/persons	**0.75**	− 0.10
7. I have difficulty complying with demands and requests from others unless they are carefully presented	**0.75**	0.02
5. I tell other people how they should behave, but do not feel these rules apply to me	**0.74**	0.03
1. I obsessively resist and avoid ordinary demands and requests	**0.72**	0.11
23. I ensure any social interaction is on my own terms	**0.72**	0.08
16. I know what to do or say to upset particular people	**0.71**	− 0.09
12. I am unaware or indifferent to the differences between myself and figures of authority (e.g. parents, teachers, and police)	**0.65**	− 0.03
22. I have periods when I have extremely emotional responses (e.g. crying/giggling, becoming furious) to what others would think small events	**0.64**	0.25
21. I sometimes use outrageous or shocking behaviour to get out of doing something	**0.62**	0.19
2. I complain about illness or physical incapacity to avoid a request or demand	**0.60**	
4. I find everyday pressures (e.g. having to go on a routine trip/visit dentist) intolerably stressful	**0.60**	0.19
13. I will still sometimes have a ‘meltdown’ (e.g. scream, tantrum, hit, or kick) if I feel pressurised to do something	**0.58**	0.27
9. I show little shame or embarrassment (e.g., I might throw a tantrum in public and not be embarrassed)	**0.54**	0.10
19. I can be distracted (preoccupied) ‘from within’ (i.e., absorbed in my own world)	**0.54**	0.26
18. I deny things I have done, even if I am caught “red handed”	**0.53**	0.24
26. I can be passive and difficult to engage	0.48	0.16
6. I mimic other people’s mannerisms and styles (e.g., use phrases adopted from other people to express myself to others)	0.44	0.38
20. I make an effort to maintain my reputation with other people	0.36	− 0.18
14. I like to be told I have done a good job	0.29	− 0.18
8. I take on roles or characters (from TV/real life) and ‘act them out’	0.10	**0.78**
10. I invent fantasy worlds or games and act them out	0.06	**0.75**
24. I prefer to interact with others in an adopted role, or communicate through props or objects	0.24	**0.58**

Factors extracted using principal component analysis, and obliquely rotated in nine iterations. Loadings over 0.5 in bold

*EDA-QA* Extreme Demand Avoidance Questionnaire (Adult) survey

##### The Autism Spectrum Quotient—Short Form (ASQ-SF; Kuenssberg et al. 2014)

The ASQ-SF is a 28-item item version of the full Autism Spectrum Quotient (ASQ; Baron-Cohen et al. 2001) and was used to quantify cognitive and behavioural features associated with ASD and examine their overlap with scores on the EDA-QA. The ASQ-SF is based on a confirmatory factor analysis (CFA) of the abridged version of the ASQ (Hoekstra et al. 2011). CFA optimises stability of solutions, and the restructuring of the ASQ-SF was based on responses from persons with ASD. The ASQ-SF has subscales of difficulties with social skills (Cronbach’s alpha = 0.85), routine (alpha = 0.48), switching (alpha = 0.53) and imagination (alpha = 0.75), and a preoccupation with patterns and numbers (“numbers”) (alpha = 0.66; all alphas from Hoekstra et al. 2011). The social subscale of the ASQ corresponds to impairments in social skills and communications diagnostic criterion of the disorder, whereas routine and numbers preferences correspond to the restricted and repetitive behaviours associated with ASD. Scoring rules for the ASQ-SF were as per Hoekstra et al. (2011), using the full response range (1–4) rather than dichotomised responses.

##### The Inventory of Callous Unemotional Traits (ICU; Kimonis et al. 2008)

The ICU is a 24-item inventory for examining antisocial behavioural traits in children and adolescents, measuring behavioural precursors to explicit personality disorder and psychopathy in younger persons (Herpers et al. 2014), for whom clinical diagnoses are less reliable (Frick and White 2008). The ICU has been widely researched in clinical and offending populations, and items are brief and straightforward. The scale comprises four dimensions; careless, callous, unemotional, and uncaring, with internal reliabilities typically in the 0.70 s (e.g., Jones et al. 2010). The scale is also associated with antisocial behaviour and psychopathology in young adults (Byrd et al. 2013).

##### Personality Inventory for DSM-5—Brief Form (PID-5-BF; Krueger et al. 2013)

The PID-5-BF is a 25-item brief self-report screening instrument which examines behaviours associated with personality disorder in terms of underlying behavioural traits: negative affect, detachment, antagonism, disinhibition, and ‘psychoticism’ (i.e., schizotypal qualities such as eccentricity, cognitive perceptual dysregulation, unusual beliefs and experiences). These dimensions form stable psychopathological traits (Fossati et al. 2017). The use of such a measure overcomes labelling difficulties associated with specific personality disorder diagnoses taken from a screening instrument, and instead focusses on the dimensionality of the behavioural traits.

#### Plan of Analysis

To test the structure of the scale, all items in the EDA-QA were entered into an item principal components analysis with oblique rotation of the emergent factors. With 347 participants and 26 items, the item-participant ratio was 13:1, optimising production of a stable solution. Parallel analysis was used to minimise over-factoring (O’Connor 2000), and indicated that eigenvalues below 1.56 should be treated with caution. A univariate scale equivalent to that identified by O’Nions et al. (2014a) was sought, with a similarly high internal reliability. This structure was tested using CFA via AMOS using two indices of fit; the comparative fit index (CFI; ideally over 0.9); and the root mean square error of approximation (RMSEA, seeking values below 0.1). The Chi square CMIN/DF was used to compare differences in fit for the two models. Inter-relationships between the EDA-QA with the other dimensions of interest were tested by means of an exploratory factor analysis that examined how the EDA-QA loaded within the factor space defined by the PID-5-BF, ICU, and ASQ-SF subscales.

### Results

#### Structure of the EDA-QA Scale

EDA-QA data were suitable for this factor analysis (Kaiser–Meyer–Olkin measure of sampling adequacy = 0.93, Bartlett’s test of sphericity χ^2^ (325) = 4073.57, p < .001). The initial analysis and pattern matrix output revealed two factors with eigenvalues over 1.56; a general EDA dimension (eigenvalue = 10.54, 40.54% variance explained), and a minor fantasy dimension comprising 3 items (eigenvalue = 1.74, 6.68% variance, total explained variance = 47.2%). The two factors correlated at 0.26, p < .001. This solution suggests the fantasy factor is a very secondary element to a general measure of self-reported adult EDA. Four scale items (26. “I can be passive and difficult to engage”; 6. “I mimic other people’s mannerisms and styles (e.g., use phrases adopted from other people to express myself to others)”; 20. “I make an effort to maintain my reputation with other people”, and “14. I like to be told I have done a good job”) did not have significant loadings on either dimension. The overall reliability (Cronbach’s alpha) of the scale was 0.94 (0.73 for the three fantasy items). The reliability of the univariate EDA-QA scale was comparable with the instrument measuring EDA from parental ratings of their children (the EDA-Q; O’Nions et al. 2014a).

A CFA of the EDA-QA items was conducted with AMOS, testing two models; a unidimensional (undifferentiated) model in which all the symptoms load onto a single factor, against a multidimensional model suggested by the exploratory factor analysis, which compared two factors. The single factor model had a CMIN of 2.408, a GFI of 0.881 (adjusted GFI = 0.853), and an RMSEA of 0.064. The two-factor model had a CMIN of 2.140, a GFI of 0.888 (adjusted GFI = 0.864), RMSEA = 0.057. The fit indices indicated the GFI was marginally acceptable, whilst the RMSEAs were both approached being a very good fit. The difference between these two models was 0.268 with 3 d.f., and not-significant (Loehlin 1987), suggesting both solutions are equally valid. For parsimony, a single factor model of the measure was adopted. A copy of the AMOS output for both of these analyses is provided in the supplementary materials.

#### Consistency of Child and Adult EDA-QA Ratings

**Fig. 1:**
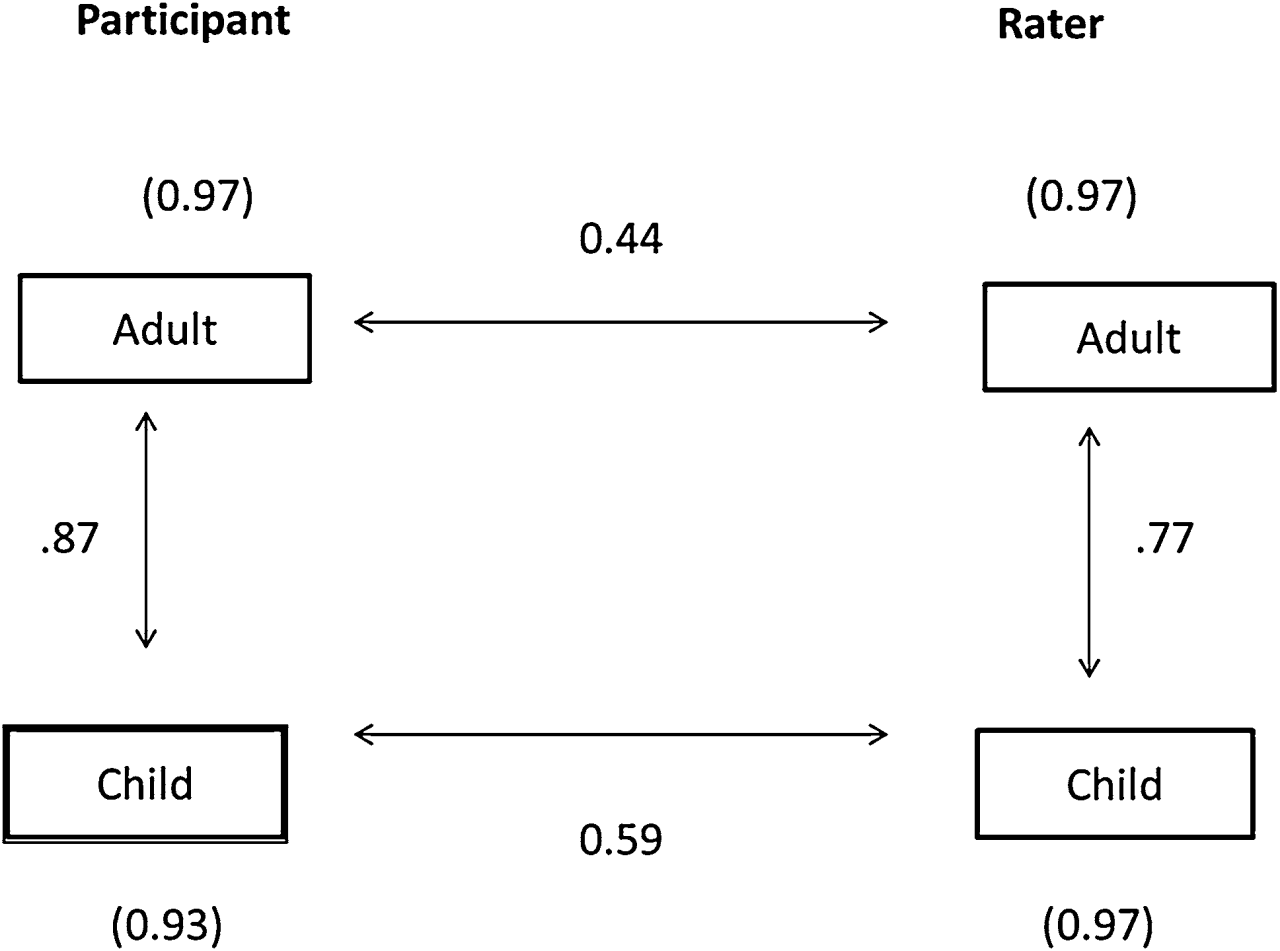
correlations between participant and informant for rated EDA-QA as a child and adult (n = 32). Panel showing the correlation between participant’s self-rating and rater’s peer rating of total score on EDA rating scale for the participant as an adult and child (Pearson’s r). Reliabilities of EDA scales (Cronbach’s alpha) in parentheses

A subset of participants (n = 32) nominated peers (mostly parents and siblings) to rate them on retrospective EDA ratings of the participant as a child, which were correlated with concurrent ratings of the participant’s behaviour (Fig. 1). Inter-rater and child–adult time point EDA-QA correlations for the individual as a child and an adult by the participant and their peer rater were all significant. The internal consistency scores (Cronbach’s alphas) shown in Fig. 1 indicate that the EDA-QA remained highly reliable.

#### Comparison of Psychometric Scores for Persons with Prior Diagnosed ASD and Those Without an ASD Diagnosis

There were 58 persons in the sample reporting a formal prior diagnosis of ASD, compared to 289 persons who did not have this diagnosis. Persons reporting an ASD diagnosis scored significantly higher on all ASQ-SF subscales and the total than persons who did not report a prior ASD diagnosis (all *t* differences p < .001 bar ASQ-SF numbers, p = .014). There was no difference between persons with self-reported “formally diagnosed” ASD and the remainder of the sample for the EDA-QA (*t* = − 0.94, p = .35; figures for score distributions provided in supplementary study materials). Comparison of the two groups for ICU and PID-5-BF found trends for persons with prior ASD to have higher negative affect and detachment (*t* = − 1.95, p = .052, and *t* = − 1.81, p = .07), and a more robust difference in PID-5-BF psychoticism (*t* = − 2.20, p = .028); however, these results did not survive correction for multiple comparisons. Thus, although persons with reported diagnosed ASD had higher ASQ-SF scores, they did not have higher EDA-QA scores, nor strong evidence of psychological distress or disturbed personality.

#### Relationship Between Other Trait Measures and the EDA-QA

Descriptive statistics for the psychometric measures of callous–unemotional traits, personality disorder traits, and ASD are presented in Table 2. While ASQ-SF routines and switching subscales had low internal consistency, the internal consistency of the total ASQ-SF was very acceptable, as were the other personality and behavioural self-report measures.

**Table 2:** Means, standard deviations, and internal reliability of observed measures

Variable	Mean	Standard deviation	Min	Max	Reliability (Cronbach’s alpha)
Child EDA-QA	38.72	17.27	2	78	0.93
Adult EDA-QA	38.48	17.72	2	78	0.94
ASQ-SF total score	76.16	12.74	36	112	0.83
ASQ-SF social skills	20.56	4.90	7	28	0.75
ASQ-SF routines	11.29	2.41	5	16	0.36
ASQ-SF switching	12.11	2.66	5	16	0.56
ASQ-SF imagination	18.84	4.95	8	32	0.66
ASQ-SF numbers	13.34	3.76	5	20	0.68
ICU careless	10.9	3.8	6	24	0.74
ICU callous	13.0	3.8	6	24	0.70
ICU unemotional	15.2	4.5	6	24	0.79
ICU uncaring	11.6	3.92	6	24	0.78
PID negative affect	14.8	3.3	6	20	0.69
PID detachment	12.6	3.3	5	20	0.67
PID antagonistic	10.3	3.7	5	20	0.76
PID disinhibition	11.7	4.1	5	20	0.83
PID psychoticism	13.6	3.8	5	20	0.78

*EDA* extreme demand avoidance, *EDA-QA* Extreme Demand Avoidance—Questionnaire, Adult, *ASQ-SF* autistic spectrum quotient—short form, *ICU* inventory of callous unemotional traits, *PID* personality inventory for DSM-5—brief form

Simple correlations between the EDA-QA total score and the ASQ-SF subscales and total score were all positive and significant, with more PDA symptoms in persons reporting more ASD features (ASQ-SF social skills: *r* = 0.26, routines: *r* = 0.37, switching: *r* = 0.36, imagination: *r* = 0.14, numbers: *r* = 0.35, and total score: *r* = 0.40. All were significant at p < .001 bar imagination, p < .009). Given the large amount of covariance between the ASQ-SF, ICU, and PID-BF-5 subscales, all trait measures plus the adult self-report EDA measure were entered into a principal components analysis with varimax rotation to examine the pattern of loadings (Table 3). Parallel analysis indicated only factors with eigenvalues < 1.37 should be accepted. Data were suitable for this type of analysis (Kaiser–Meyer–Olkin measure of sampling adequacy = 0.81, Bartlett’s test of sphericity χ^2^ (105) = 2061.05, p < .001). Three varimax-rotated factors were generated in seven iterations, which explained 56.7% of the variance (rotated eigenvalues 2.94, 2.80, and 2.77, successively). Loadings of 0.5 or above between the variable and the resultant rotated factors were used to assist interpretation.

**Table 3:** Principal components analysis (with varimax rotation) of psychometric measures in sample (n = 346)

	F1	F2	F3
ICU callous	**0.82**	0.21	0.13
ICU careless	**0.63**	0.19	− 0.04
Antagonism	**0.60**	**0.51**	− 0.09
Detachment	**0.51**	0.07	**0.50**
ICU uncaring	**0.84**	0.06	0.24
Negative affect	0.05	**0.82**	0.20
EDA-QA	0.40	**0.73**	0.25
Psychoticism	0.25	**0.70**	0.26
Disinhibition	0.41	**0.62**	− 0.04
SAQ social skills	0.00	0.09	**0.79**
SAQ routines	0.04	0.18	**0.69**
SAQ imagination	0.11	− 0.02	**0.61**
SAQ switching	− 0.11	0.40	**0.53**
SAQ numbers	0.10	0.15	**0.52**
ICU unemotional	0.35	− 0.40	0.49

Rotation converged in seven iterations. Loadings over 0.50 in bold

*EDA-QA* self-report adult extreme demand avoidance survey, *ASQ-SF* autistic spectrum quotient—short form, *ICU* inventory of callous unemotional traits, *PID* personality inventory for DSM-5—brief form

The first factor comprised many of the hostile behaviours: ICU uncaring, ICU Callousness, ICU carelessness, PID Antagonism, and PID detachment. The EDA-QA did not load at the minimal criterion for this dimension. The second dimension contained the highest loading for the EDA-QA, and was defined by greater association with PID Antagonism, PID negative affect, PID psychoticism, and PID disinhibition—four of the five Krueger PID-5 personality disorder indicators. ASQ-SF switching was the highest ASD subscale loading for the EDA-QA dominated factor, but at 0.40 was below the criterion for factor interpretation. The third dimension comprised a clear ASD factor, with positive loadings for ASQ-SF social skills, routines, imagination, switching, and numbers, and PID detachment, with a loading of 0.49 for ICU unemotionality. These results suggest that EDA-QA self-reports are most strongly associated with antagonism and extreme emotionality/emotional disinhibition (characteristics associated with personality disorder), and to a lesser extent scores on overt hostility and autism-related factors.

#### Demographic and Clinical Associations with EDA-QA

To examine general demographic associations with the EDA-QA, a multiple regression was conducted in which gender, age, years of education, occupational status, and prior formal mental health diagnosis were all predictors. The overall regression was significant but of small effect: R = 0.26, adjusted R^2^ = 0.06, F(5, 327) = 4.912, p < .001. Of the individual predictors, three were independently significant; male gender (standardised beta = − 0.132, *t* = − 2.49, p < .015); fewer years of education (standardised beta = − 0.138, *t* = 2.54, p < .011), and having a prior mental health diagnosis (standardised beta = 0.165, *t* = 3.06, p < .002).

### Discussion (Study 1)

Study 1 describes the first attempt to quantify self-identified PDA traits in adults using the newly developed EDA-QA and explores the relationship between PDA traits and other dimensions of psychopathology. Results indicate that the EDA-QA is internally reliable and consistent with peer ratings for adults who span a range in terms of self-identified behaviours described in children with putative PDA. CFA fitness indices were adequate to good, though further work is required with a range of data sets and more explicitly clinical cohorts to refine measurement. Nevertheless, a brief self-report measure to operationalise adult PDA will enable further research on the condition. In principle, EDA-QA and ASD scores were associated, though a more rigorous analysis suggested that self-identified PDA traits loaded most strongly onto a dimension encompassing four of the five PID-5 traits; Antagonism, negative affect, psychoticism, and disinhibition. In adults, these traits and behaviours are associated with personality disorder diagnoses (Al-Dajani et al. 2016). This is compatible with behavioural and subjective reports of extreme emotional distress and poor behavioural regulation in children, adolescents, and adults with reported PDA.

The factor analysis revealed that self-reported PDA traits had a marginal (0.40) loading on the overt hostility dimension and loaded below the significance criterion for the autism dimension (0.25). A marginal ASQ loading (0.40) for the (unreliable) switching subscale was the best ASD-related symptom for the EDA-loaded factor, One explanation for the weaker association between PDA and ASD features could be that, in a community cohort, PDA is tapping variance related to a general psychopathology or a ‘p’ factor (Caspi et al. 2014); which is distinct from specifically ASD-related and hostile traits, but not in itself separable from other ‘p’-related dimensions. Indeed, recent work suggests that a large general latent variable underlies many different expressions of psychopathology (Lahey et al. 2017).

To date, research on PDA in young people has predominantly focused on the behaviours in the context of ASD (e.g. O’Nions et al. 2016; Gillberg et al. 2015). However, similar behaviours may be seen in other groups. The concept of equifinality highlights that a particular higher-level behaviour may have different drivers in different individuals (Cicchetti and Rogosch 1996). PDA behaviours may relate to ASD via general psychopathological difficulties rather than ASD itself. In the context of ASD, extreme emotionality may reflect hyper-sensitivity to deviations from expected events, rigid cognitive processing, or aberrant processing of social cues. The present findings suggest that more investigation of PDA behaviours in broader samples is warranted, particularly in profiles associated with extreme emotional dysregulation (e.g., adult personality disorders). However, our findings indicate that the EDA-QA is a reliable measure capturing the self-identification of constructs described in the observer-rated PDA measure for children, and that other behavioural dimensions (e.g. disinhibition, negative affect) relate to these features. Study 1 provides useful insight into the relationship between adult PDA and other dimensions of psychopathology. However, some limitations should be noted. First, ASD was assessed using a brief measure (the ASQ-SF), which predominantly focusses on the more rigid and systematising aspects of ASD, as opposed to social features. More extensive coverage of social aspects of ASD may reveal a stronger relationship between EDA and ASD traits. A second limitation is that the theoretical model for assessing personality was restricted to personality disorder symptoms, as opposed to general personality dimensions that capture individual differences in population samples more sensitively. Extant research suggests general personality dimensions relate to ASD traits. For example, Austin (2005) found persons higher on the ASQ and a brief screen for Asperger’s syndrome lower in extroversion and agreeableness, and higher in neuroticism (i.e., emotional instability). Jones et al. (2011) observed that associations between lower agreeableness, lower conscientiousness and higher neuroticism routinely emerge in association with antisocial behaviour and aggression. Measuring general personality dimensions may help to uncover contributions to variance in PDA.

Study 2 sought to expand on the findings of Study 1 on the EDA-QA by assessing persons using the full (rather than short-form) ASQ, gathering data on the ASQ’s associated empathising measure, and assessing general personality disposition. In addition, Study 2 explored the predictive relationship between these dimensions and offending behaviour, quantified using a measure of self-reported delinquency. Studies suggest that, given anonymity, self-reported offending can be reliably measured (Thornberry and Krohn 2000).

We predicted that persons with higher scores on the EDA-QA would have lower agreeableness, lower conscientiousness, greater neuroticism, lower extroversion, lower empathy, and would have committed more delinquent acts. Lastly, we sought to examine whether a measure of ASD with more extensive coverage of social components would indicate a stronger association with the EDA-QA than found in Study 1.

Because many of our predictor variables in Study 2 were inter-correlated, path analysis was used to examine relationships between our variables of interest. This also allowed us to examine the predictive relationship between PDA and offending behaviour, over and above the other measured dimensions.

## Study 2: Validation of the EDA-QA in Relation to Fuller Measures of ASQ, EQ, Personality, and Offending. Method and Procedure

Data were gathered from a population sample and recruited from sources where some individuals self-identified as having ASD or PDA features. Like Study 1, an online questionnaire was used to collect data from the general population to optimise sampling.

### Participants

Given an estimated effect size of 0.9, a sought statistical significance of p = .01 and power of 0.95, this study required a minimum of 175 participants. The final sample consisted of 191 participants [47 males, 14 females, 3 other (self-declared ‘non-binary/gender fluid’)], mean age 29.15 years (SD = 13.15 years, range 18–76). All participants were proficient in English. The research link was posted on websites, including the Hanover on-line psychological research, and PDA and “Neurotypical” Facebook pages. The sample were predominantly high functioning; demographic information indicated that 83.2% had completed more than 13 years of education; 39.3% were still in full or part time study; 51.8% were currently in full or part time employment; and 8.9% were unemployed/retired. Information was collected about psychological diagnoses, both confirmed and self-identified: 26.2% reported a confirmed diagnosis (most prevalently joint anxiety and depression), and a further 11.5% suspected a present underlying condition (mostly anxiety and Asperger’s syndrome). Finally, participants were asked to indicate if they had ever been cautioned, arrested, charged, and/or convicted of any offences; 89.6% stated they had not received any of these penalties, irrespective of self-reported delinquency.

### Measures

#### The EDA-QA

This novel measure was used as described in the method and results for Study 1 above. For Study 2 the EDA-QA had an internal reliability of 0.92.

#### The Autism Spectrum Quotient (ASQ; Baron-Cohen et al. 2001)

The ASQ is a non-diagnostic test to assess ASD traits in the general population. The subscales measure communication (Cronbach’s alpha = 0.65), social skills (0.77), imagination (0.65), local details (0.63), and attention switching (0.67; internal consistency scores from Baron-Cohen et al. 2001). The measure is scored on a 4-point scale with forced-choice statements. Scores are summed for an overall total. Baron-Cohen et al. (2001) propose that a score above 32 point denotes a very high likelihood of ASD being present, but as the ASQ is not a diagnostic test, clinical cut-offs should be read with caution. In Study 2, the full ASQ was administered. Analysis of the ASQ in a large non-clinical sample suggested the measure had a three, rather than five factor solution: ASQ social skills, ASQ numbers/details/patterns, and ASQ communications/mindreading (Hurst et al. 2007). This scoring procedure was adopted here. In addition, as the full ASQ was being used, it was also possible to classify individuals in terms of meeting possible ASD, namely scoring 32 or more on the full ASQ (Baron-Cohen et al. 2005). There were 13 persons in the cohort who had caseness for the criterion on the ASQ, compared to 176 below.

#### The Empathy Quotient (EQ; Baron-Cohen and Wheelwright 2004)

The EQ examines empathy levels in individuals (Cronbach’s alpha = 0.79). The measure is scored on a 4-point scale with forced-choice statements where some ‘slightly agree’ responses score 1 point, and ‘definitely agree’ responses score 2 points; reversed items score 1 point on ‘slightly disagree’ responses, and 2 points on ‘definitely disagree’ responses. Scores are summed for an overall measure. In the current study we were not concerned with cut-off or impairment scores for this measure.

#### The 50-Item Big-Five Factor Scale of Personality (IPIP-50; Goldberg 1999)

The IPIP-50 assesses five domains of personality (agreeableness, extraversion, emotional stability, intellect-imagination and conscientiousness). Scale Cronbach’s alpha values are 0.82, 0.87, 0.86, 0.84 and 0.79 respectively (Goldberg 1999). The IPIP-50 is scored on a 5-point Likert scale with responses to self-describing statements ranging from 1 ‘very inaccurate’ to 5 ‘very accurate’.

#### The Self-Report Early Delinquency Scale (SRED; Moffitt and Silva 1988, Adapted by; Charles and Egan 2005)

The SRED quantifies past antisocial and delinquent behaviours. It has an overall Cronbach’s alpha value of 0.70. The SRED asks respondents to state whether they have never (1), once (2), or more than once (3) tried a particular act. There are five SRED subscales (antisocial; transgressive; criminal behaviours; trouble through alcohol and vandalism; and acquisitive acts). Numerical response values were summed to give an overall score.

#### Procedure

As with Study 1, ethical approval was gained from the University’s Medical School. The survey was advertised on social media, requesting participants complete the survey via a link hosted by bristolonlinesurveys.ac.uk. Participants were given information about the purpose of the study, what was expected of them, and gave informed consent. Anonymity was ensured by not gathering uniquely identifying details.

#### Plan of Analysis

We examined whether full scores on the ASQ were more informative as to associations with the EDA-QA than the shorter measure, compared persons meeting putative caseness for those above and below the ASD ASQ criterion on the EDA-QA, and tested whether one could predict general delinquency from the EDA-QA, taking into account the correlations between ASQ, EQ, and general personality. The latter analysis was conducted using path analysis calculated using AMOS (Arbuckle and Wothke 2003), and confirmed using regression.

### Results

Correlations between EDA-QA, ASQ, EQ, personality, and self-reported delinquency measures, and their associated summary statistics and reliabilities are presented in Table 4. Most measures were highly reliable, with the EDA-QA having a Cronbach’s alpha of 0.92, very similar to that observed in Study 1. While the ASQ social skills subscale was internally reliable, ASQ numbers patterns and details, and ASQ Communication and Mind Reading subscales were less so. Given these poorer ASQ subscale reliabilities, we took the highly reliable total ASQ as an index of ASD symptoms, and do not report subscale associations.

**Table 4:** Pearson’s r correlation between ASQ, EDA, EQ, IPIP, and SRED (n = 187)

	EDA-QA	EQ	E	A	C	ES	I	SRED
ASQ total (0.85)	0.49**	− 0.60**	0.07	− 0.53**	− 0.18	− 0.47**	− 0.09	0.02
EDA-QA	(0.92)	− 0.36**	− 0.09	− 0.42**	− 0.26**	− 0.50**	0.00	0.34**
EQ		(0.87)	0.02	0.73**	0.31**	0.24**	0.28**	− 0.10
(Cronbach’s alpha)			(0.89)	(0.84)	(0.84)	(0.90)	(0.87)	(0.92)

All correlations over 0.24, p < .001 and marked**; internal alpha reliabilities inside parentheses

*ASQ total* full Autism Spectrum Questionnaire total score, *EDA-QA* extreme demand avoidance—self report for adults, *EQ* Empathy Questionnaire, *E* extroversion, *A* agreeableness, *C* conscientiousness, *ES* emotional stability, *I* intellect, *SRED* self-report early delinquency total (log10 transformation)

During exploration of the distributions of our measures, we identified two persons with very high SRED scores relative to the rest of the cohort exaggerating the measure’s kurtosis; a log 10 transformation of the SRED reduced this kurtosis to an acceptable level. Simple correlations indicated higher scores on the EDA-QA were associated with more ASQ symptoms, lower agreeableness, lower emotional stability, and higher overall self-reported delinquency (all coefficients p < .001). ASQ and EQ measures also showed significant associations with one another and personality traits. Persons meeting probable caseness for ASD on the ASQ had significantly higher EDA-QA scores, and significantly lower EQ, agreeableness, and emotional stability measures compared to those below the ASQ criterion [*t* = − 3.18 (p = .002), 6.40, 5.29 (both p = .001), and 3.17 (p = .006), respectively].

An exploratory path analysis was conducted on the psychometric measures using the total score on the ASQ as an index of ASD. This is a better use of information than dichotomising ASQ scores, as the full range of information is used, including borderline scores which would be otherwise regarded as representing an absence of the quality when the quality is clearly present to a considerable degree. Path analysis is a type of structural equation model (a technique that combines factor analysis and multiple regression) to describe structural relations between measured variables (Byrne 2016).

**Fig. 2:**
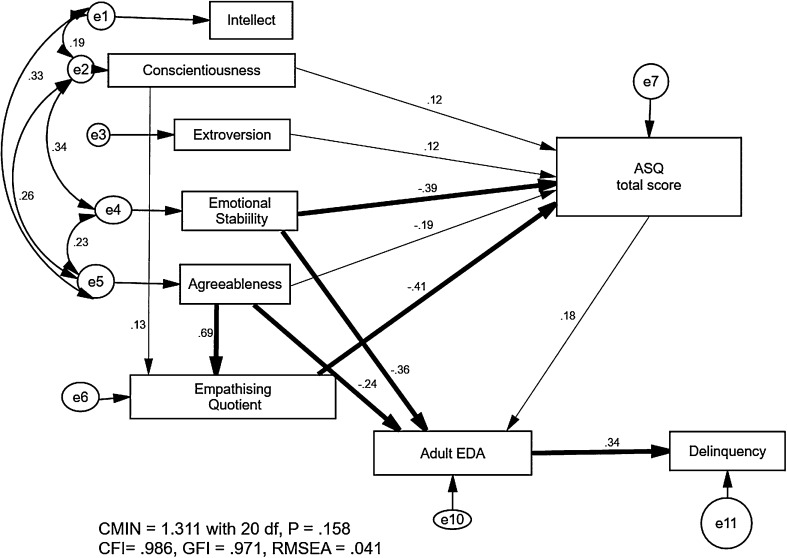
Path analysis fitting EDA-QA to EQ, ASQ and personality to delinquency (Study 2). Exploratory path analysis model calculated with AMOS. Measured variables are shown in boxes. Circles with an e and a number are error variances. Double-headed arrows indicated covariance between error variances. Thin solid arrow pathways are significant standardised regression coefficients significant at p < .02 or below, thick arrows at p < .001. Goodness of fit indicators (CMIN, CFI, GFI, RMSEA) are all excellent. *ASQ* Autism Spectrum Questionnaire, *EDA* extreme demand avoidance-QA

The path analysis fitted the EDA-QA, the total ASQ score, the EQ, and the IPIP-50 personality dimensions to a specific outcome, total SRED delinquency (Fig. 2). Significant pathways are indicated, with the heavier arrowed lines indicating they are significant at p < .001; the numbers associated with the arrowed pathways are standardised regression coefficients. There was a significant direct path from the total ASQ variable to EDA-QA (critical ratio (CR) = 2.44, p = .015). The EDA-QA score was also predicted by lower agreeableness (CR = − 3.56, p = .001), and lower emotional stability (CR = − 5.50, p = .001). These results suggest that in a predominantly female community sample, EDA relates to both ASD traits and personality features. EDA directly predicted delinquency, with a path loading of 0.34 (CR = 4.93, p = .001). The data in the model also enabled a representation of how personality and the EQ relate to the ASQ. The total ASQ score was predicted by lower emotional stability, a lower EQ, lower agreeableness, and slightly great extroversion and conscientiousness [CR = − 7.12, − 5.44 (both p = .001), − 2.52, 2.47, and 2.23 (p = .012, .02, and .026), respectively]. Finally, the model indicated that the EQ was strongly predicted by agreeableness (CR = 13.65, p = .001) and conscientiousness (CR = 2.64, p = .018), The overall model fitted the data extremely well (CMIN = 1.1311 with 20 d.f., CFI = 0.99, Goodness of fit index = 0.97, root mean square error of approximation = 0.041). This model suggests a structural arrangement of constructs plausibly related to the underpinnings of adult EDA traits in a community sample consisting predominantly of females.

As a final test of the incremental value of PDA assessment for predicting delinquency, a stepwise linear regression was calculated, entering all 5 IPIP personality dimensions as a block, then the ASQ, and finally the EDA-QA. Unlike the ASQ, entry of personality and EDA caused a significant F change (p = .017, n.s., and p < .001, successively), the EDA-QA had the greatest incremental value (F(7, 181) = 29.65, p < .001). The final model was R = 0.451, adjusted R^2^ = 0.173, F(7, 181) = 6.609, p < .001. Only one variable significantly predicted delinquency in the final model; EDA-QA (standardised beta weight = β = 0.461, p < .001); all the personality and ASQ variance was captured by the EDA-QA when predicting the overall outcome.

### Discussion (Study 2)

The second study examined the EDA-QA in a community sample and measured ASD traits more thoroughly, using the full ASQ. It also measured general personality dimensions suitable for studying traits in community samples, rather than indications of gross behavioural disorder. In addition, the EQ was used to measure empathising. Lastly, a checklist of antisocial acts used in forensic psychological research sampled the range of offences that participants had committed. The EDA-QA again showed excellent reliability.

A path analysis to fit the data indicated that ASQ and EDA-QA scores were positively related. Both also related to personality, with total ASQ being significantly associated with a lower EQ, lower agreeableness, lower emotional stability, and, to a lesser extent, with greater Conscientious and agreeableness. The EDA-QA measure was associated with lower agreeableness, lower emotional stability, and higher scores on the ASQ. The effects were stronger for personality traits than for ASQ scores, suggesting it may be personality that differentiates how ASD traits are expressed, with more emotionally unstable and antagonistic persons with ASD expressing PDA-type qualities. Research will show whether persons with low emotional stability and antagonism may likewise present with PDA symptoms despite not having significant ASD features. In short, in community samples, is possible that PDA captures general p-factor psychopathology features (Caspi et al. 2014). In the context of ASD, PDA may reflect a developmental consequence of anxiety surrounding routine demands emerging in response to ASD-related vulnerabilities (e.g. sensory sensitivities, anxiety about uncertainty, or other emotive stimuli).

## Overall Discussion

These two well-powered studies provide good evidence for the reliability and validity of the EDA-QA as a useful tool to assess self-identified traits of relevance to the profile described by Newson et al. (2003). Our findings suggest that in a community sample, self-reported PDA traits partially relate to self-reported ASD traits. However, both studies were conducted in adults recruited from the community who did not necessarily report symptoms of psychopathology, and a substantial proportion of participants were female. It remains possible that different patterns of association would arise if the EDA-QA was deployed within a cohort closer to that driving the need for PDA research, with a higher incidence of diagnosed ASD and/or more co-existing psychopathology; self-reported ASD is not without it’s difficulties (Bishop and Seltzer 2012). Notably, the samples for both studies over-represented females; excluding persons who rated their gender “other”, the standardised sex ratio for Study 1 was 40.87, and Study 2, 33.81. Nevertheless, no significant differences were found for EDA-QA scores for males vs. females (*t* values < 1.83, n.s.). Supplementary Figs. 3 and 4 also illustrate the lack of gender effects on the EDA-QA.

This study provides a means to extend research on PDA into adult samples, and to examine, in field settings, PDA traits in more narrowly-defined formal clinical cohorts with DSM-5 diagnoses. The self-report and observer-rated forms of the EDA instrument for adults have internal and peer-rated reliability, and are easily administered to patients, carers, and practitioners. The EDA-QA’s correlations with personality measures of antagonism and emotional instability indicate convergent validity, whilst it’s lack of association with extroversion and intellect indicates discriminant validity. The instrument could be easily integrated into assessment packages currently used with prisoners, mentally disordered offenders, and homeless people, where PDA may be suspected. A short, easy, and reliable instrument is necessary when participant patience for lengthy assessments is slight. We note, however, that one of the inherent difficulties studying a person with PDA is that they may be resistant to doing things asked of them, or to complete a research protocol where they have to abide by the rules and structures imposed by the researcher. Thus, this scale may be challenging to administer with persons in a clinical context who express more extreme presentations, lack adaptive skills or self-awareness, or are unwilling to comply and engage with research.

Persons in closed settings or with particularly acute apparent PDA may be designated as non-compliant or demand-avoidant, despite the person’s withdrawing engagement being the only expression of autonomy that they have (Clements and Zarkowska 2000). In such cases, labeling the individual as demand avoidant arguably reflects the needs of the person with authority over the ‘demand-avoiding’ individual. Given the avoidant person may be high in features of ASD, there may be mutual incomprehension regarding a need to communicate or engage in particular activities (the ‘double empathy issue’, Milton 2012). Improving communication with persons who express PDA may be critical for more constructive and ethical engagement and assessment. From the perspective of the ASD individual, avoiding demands may represent an effective way of functioning when an asocial behavioural strategy is adopted to optimize individual rather than group survival (Reser 2011).

We conclude that the EDA-QA provides a solid methodological platform for more systematic research into adult PDA in a variety of contexts, particularly clinical ones. Such research could help improve the care and management of persons who self-identify as having PDA traits, seem unable to adapt to demands placed on them by adult life, and commit acts that lead them to be involved with the CJS or mental health services.

## Electronic supplementary material

Below is the link to the electronic supplementary material.

Supplementary material 1 (DOCX 279 KB)

Supplementary material 2 (DOCX 51 KB)

Supplementary material 3 (DOCX 54 KB)
