# Supplementary material for: The Measurement of Adult Pathological Demand Avoidance Traits
Source: J Autism Dev Disord. 2018 Aug 23;49(2):481–94. doi: 10.1007/s10803-018-3722-7 (PMC6373319; doi:10.1007/s10803-018-3722-7)

Supplementary figures for “THE MEASUREMENT OF ADULT EXTREME DEMAND AVOIDANCE TRAITS” (JADD-D-17-00945).

List of contents

Fig S1: Study 1. Distribution of ASQ-SF scores for persons with formal diagnosis of ASD.

Fig S2: Study 1. Distribution of EDA scores for persons with formal diagnosis of ASD.

Fig S3: study 1. Comparison of EDA scores across group, by gender.

Fig S4: study 2. Comparison of EDA scores across group, by gender.

Fig S5: Study 1. Correlation between ASQ-short form and EDA-QA total scores.

Fig S6: Study 2. Correlation between Full ASQ and EDA-QA total scores.

Fig S7: Study 2. Correlation between EDA-QA and log10 SRED score.

Fig 1: Study 1. Distribution of ASQ-SF scores for persons with formal diagnosis of ASD.


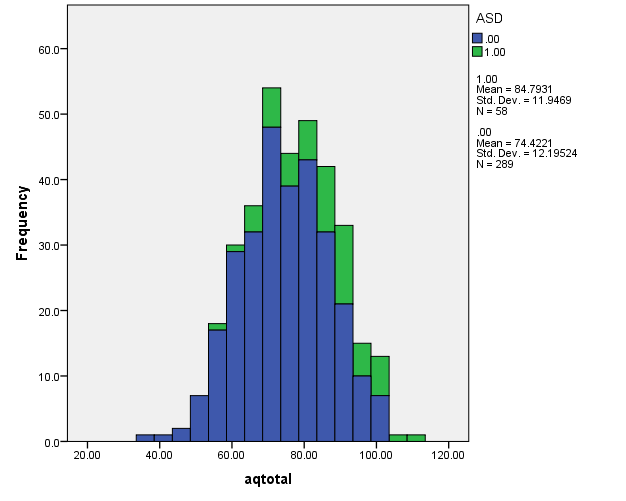


ASD 1 (persons with a formal diagnosis of ASD) have a group total score for ASQ-SF (in green) which is significantly higher than ASD 0 (no diagnosis, coloured blue): t = -5.93, P = 0.001.

Figure 2 – supplementary: Study 1. Distribution of EDA scores for persons with formal diagnosis of ASD.


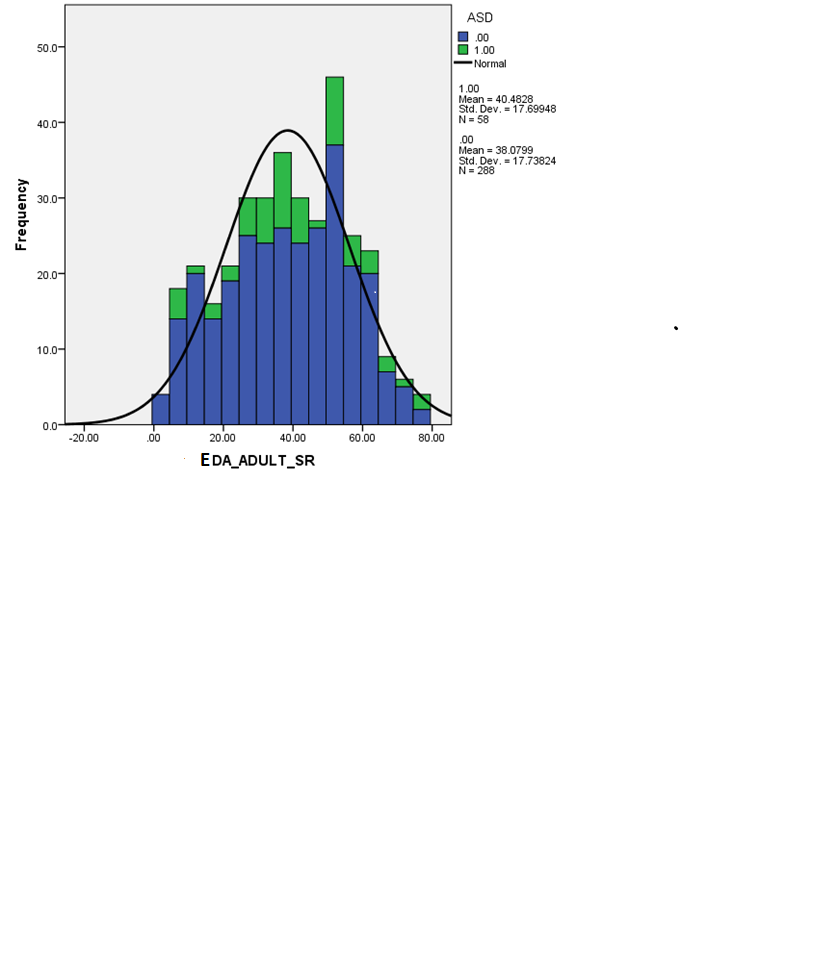


There is no difference in EDA-QA scores for ASD 1 (persons with a formal diagnosis of ASD, in green) compared to ASD 0 (no diagnosis, coloured blue): t = -0.94, n.s.

Figure 3 – supplementary. Study 1- Graph showing EDA-QA scores (mean and standard deviation) by gender.


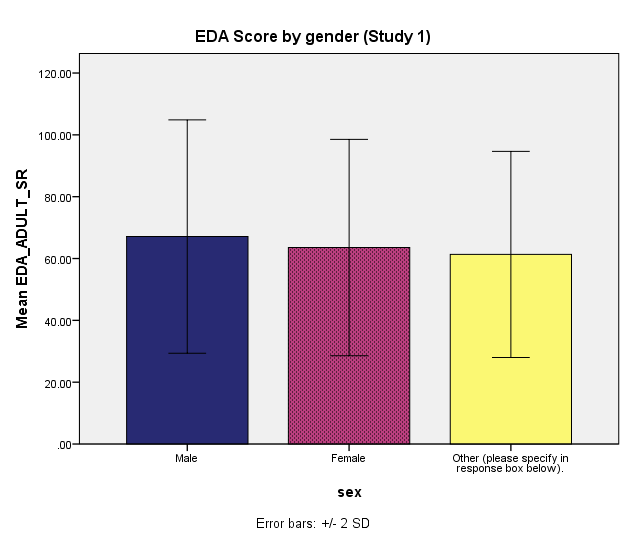


| **Descriptive Statistics** | | | |
| --- | --- | --- | --- |
| Dependent Variable: EDA_ADULT_SR | | | |
| sex | Mean | Std. Deviation | N |
| Male | 67.1064 | 18.87450 | 94 |
| Female | 63.5522 | 17.49371 | 230 |
| Other (please specify in response box below). | 61.3333 | 16.66980 | 18 |
| Total | 64.4123 | 17.87533 | 342 |

No difference between males, females, and other (transgender and related variants): F(2, 339) = 1.607, n.s.

Figure 4 – supplementary. Study 2- Graph showing EDA-QA scores (mean and standard deviation) by gender.


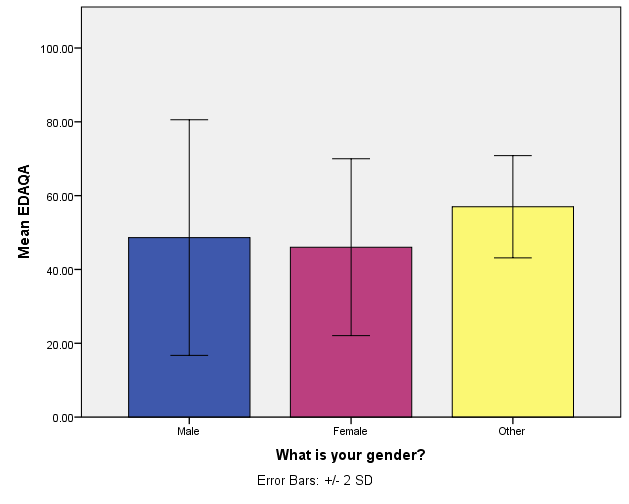


| **Descriptives** | | | | | |
| --- | --- | --- | --- | --- | --- |
| EDAQA | | | | | |
|  | N | Mean | Std. Deviation | Std. Error |  |
|  |  |  |  |  |  |
| Male | 47 | 48.6383 | 15.95568 | 2.32738 |  |
| Female | 140 | 46.0286 | 11.97536 | 1.01210 |  |
| Other | 3 | 57.0000 | 6.92820 | 4.00000 |  |
| Total | 190 | 46.8474 | 13.07175 | .94832 |  |

No difference between males, females, and other (transgender and related variants): F(2, 187) = 1.631, n.s.

Figure 5: Correlation between ASQ-short form and EDA-QA total scores (study 1).


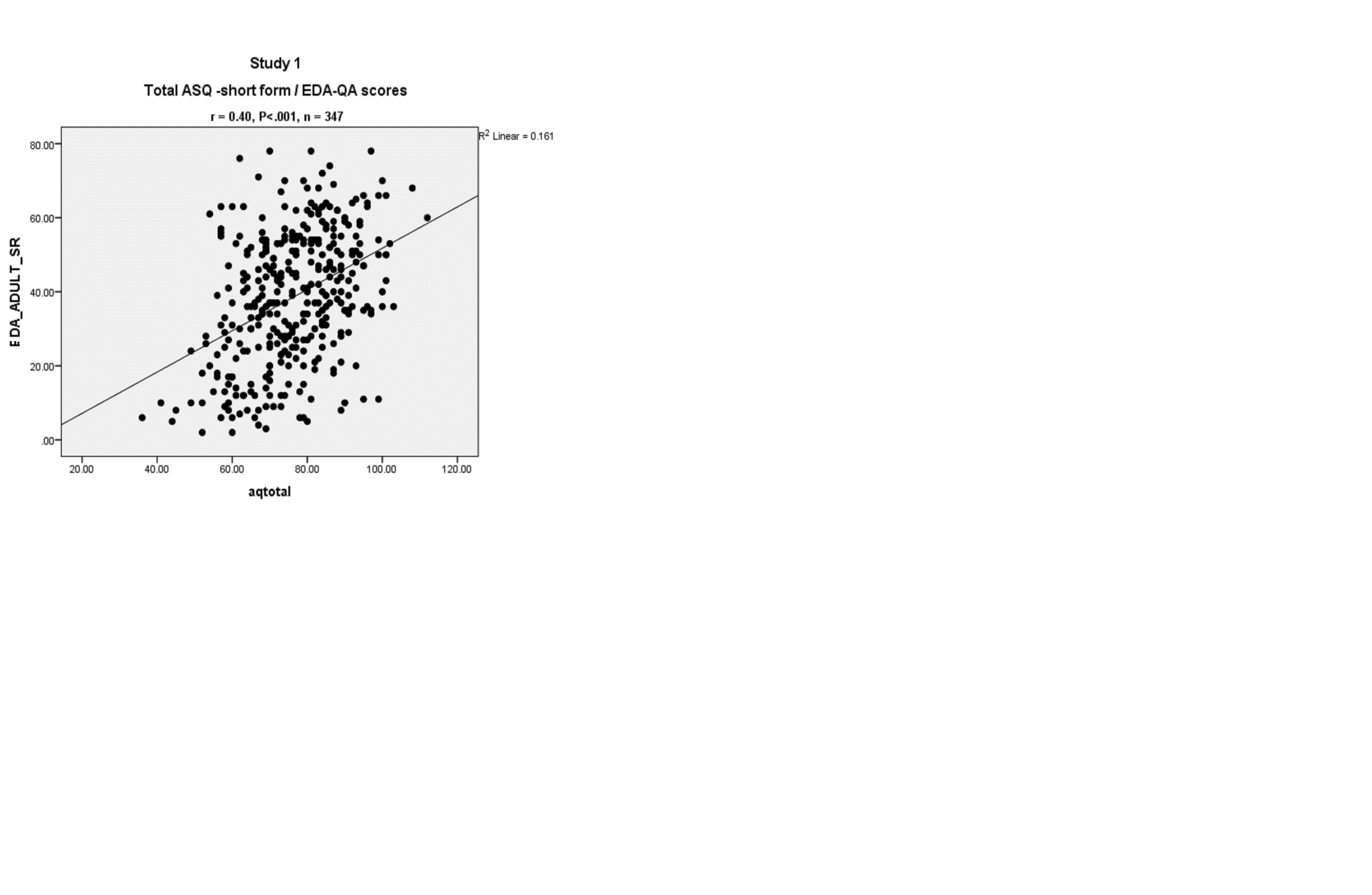


Figure 6: Correlation between Full ASQ and EDA-QA total scores (study 2).


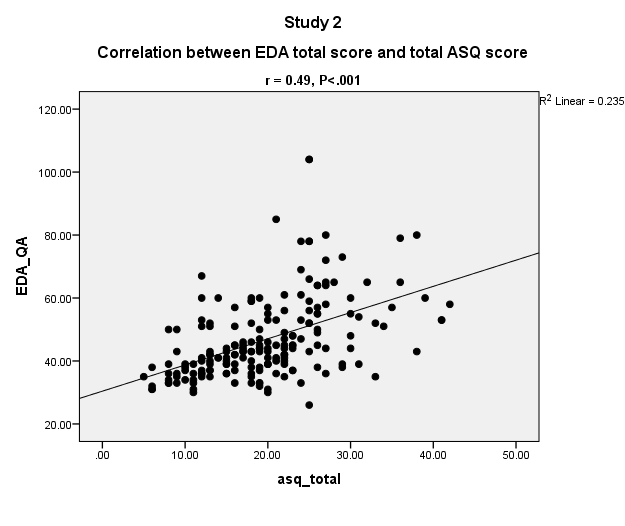


Figure 7: Study 2 – Correlation between EDA-QA and log10 SRED score.


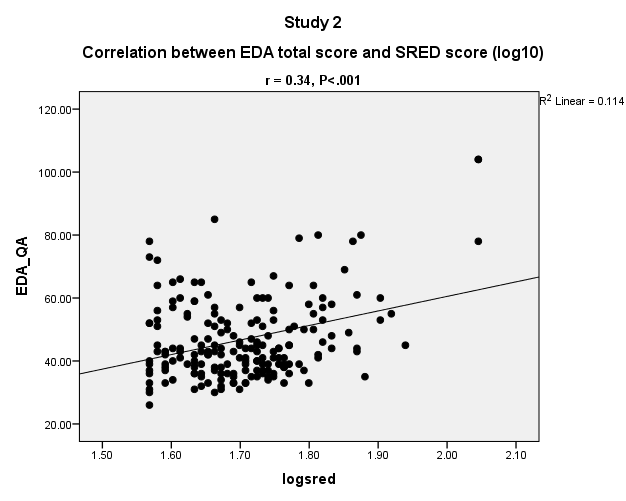

Supplement: Supplementary file 1 — Supplementary material 1 (DOCX 279 KB) [file 10803_2018_3722_MOESM1_ESM.docx]
