# Supplementary material for: The Measurement of Adult Pathological Demand Avoidance Traits
Source: J Autism Dev Disord. 2018 Aug 23;49(2):481–94. doi: 10.1007/s10803-018-3722-7 (PMC6373319; doi:10.1007/s10803-018-3722-7)
Supplement: Supplementary file 3 — Supplementary material 3 (DOCX 54 KB) [file 10803_2018_3722_MOESM3_ESM.docx]

**2 factor cfa of EDA-QA**

**Analysis Summary**

**Date and Time**

Date: 07 June 2018

Time: 18:56:14

**Title**

2 factor cfa of EDAQA: 07 June 2018 18:56

**Groups**

**Group number 1 (Group number 1)**

**Notes for Group (Group number 1)**

The model is recursive.

Sample size = 347

**Variable Summary (Group number 1)**

**Your model contains the following variables (Group number 1)**

Observed, endogenous variables

EDAQA11

EDAQA25

EDAQA15

EDAQA3

EDAQA17

EDAQA7

EDAQA5

EDAQA1

EDAQA23

EDAQA16

EDAQA12

EDAQA22

EDAQA21

EDAQA2

EDAQA4

EDAQA13

EDAQA9

EDAQA19

EDAQA18

EDAQA26

EDAQA6

EDAQA8

EDAQA10

EDAQA24

Unobserved, endogenous variables

F1

F2

Unobserved, exogenous variables

e1

e2

e3

e4

e5

e6

e7

e8

e9

e10

e12

e13

e14

e15

e16

e17

e18

e19

e20

e21

e22

e23

e24

e25

ef1

ef2

**Variable counts (Group number 1)**

| Number of variables in your model: | 52 |
| --- | --- |
| Number of observed variables: | 24 |
| Number of unobserved variables: | 28 |
| Number of exogenous variables: | 26 |
| Number of endogenous variables: | 26 |

**Parameter Summary (Group number 1)**

|  | Weights | Covariances | Variances | Means | Intercepts | Total |
| --- | --- | --- | --- | --- | --- | --- |
| Fixed | 50 | 0 | 0 | 0 | 0 | 50 |
| Labeled | 0 | 0 | 0 | 0 | 0 | 0 |
| Unlabeled | 0 | 27 | 26 | 0 | 0 | 53 |
| Total | 50 | 27 | 26 | 0 | 0 | 103 |

**Models**

**Default model (Default model)**

**Notes for Model (Default model)**

**Computation of degrees of freedom (Default model)**

| Number of distinct sample moments: | 300 |
| --- | --- |
| Number of distinct parameters to be estimated: | 53 |
| Degrees of freedom (300 - 53): | 247 |

**Result (Default model)**

Minimum was achieved

Chi-square = 528.548

Degrees of freedom = 247

Probability level = .000

**Group number 1 (Group number 1 - Default model)**

**Estimates (Group number 1 - Default model)**

**Scalar Estimates (Group number 1 - Default model)**

**Maximum Likelihood Estimates**

**Regression Weights: (Group number 1 - Default model)**

|  |  |  | Estimate | S.E. | C.R. | P | Label |
| --- | --- | --- | --- | --- | --- | --- | --- |
| EDAQA11 | <--- | F1 | 1.000 |  |  |  |  |
| EDAQA25 | <--- | F1 | 1.000 |  |  |  |  |
| EDAQA15 | <--- | F1 | 1.000 |  |  |  |  |
| EDAQA3 | <--- | F1 | 1.000 |  |  |  |  |
| EDAQA17 | <--- | F1 | 1.000 |  |  |  |  |
| EDAQA7 | <--- | F1 | 1.000 |  |  |  |  |
| EDAQA5 | <--- | F1 | 1.000 |  |  |  |  |
| EDAQA1 | <--- | F1 | 1.000 |  |  |  |  |
| EDAQA23 | <--- | F1 | 1.000 |  |  |  |  |
| EDAQA16 | <--- | F1 | 1.000 |  |  |  |  |
| EDAQA12 | <--- | F1 | 1.000 |  |  |  |  |
| EDAQA22 | <--- | F1 | 1.000 |  |  |  |  |
| EDAQA21 | <--- | F1 | 1.000 |  |  |  |  |
| EDAQA2 | <--- | F1 | 1.000 |  |  |  |  |
| EDAQA4 | <--- | F1 | 1.000 |  |  |  |  |
| EDAQA13 | <--- | F1 | 1.000 |  |  |  |  |
| EDAQA9 | <--- | F1 | 1.000 |  |  |  |  |
| EDAQA19 | <--- | F1 | 1.000 |  |  |  |  |
| EDAQA18 | <--- | F1 | 1.000 |  |  |  |  |
| EDAQA26 | <--- | F1 | 1.000 |  |  |  |  |
| EDAQA6 | <--- | F1 | 1.000 |  |  |  |  |
| EDAQA8 | <--- | F2 | 1.000 |  |  |  |  |
| EDAQA10 | <--- | F2 | 1.000 |  |  |  |  |
| EDAQA24 | <--- | F2 | 1.000 |  |  |  |  |

**Standardized Regression Weights: (Group number 1 - Default model)**

|  |  |  | Estimate |
| --- | --- | --- | --- |
| EDAQA11 | <--- | F1 | .667 |
| EDAQA25 | <--- | F1 | .692 |
| EDAQA15 | <--- | F1 | .715 |
| EDAQA3 | <--- | F1 | .663 |
| EDAQA17 | <--- | F1 | .653 |
| EDAQA7 | <--- | F1 | .698 |
| EDAQA5 | <--- | F1 | .692 |
| EDAQA1 | <--- | F1 | .704 |
| EDAQA23 | <--- | F1 | .714 |
| EDAQA16 | <--- | F1 | .642 |
| EDAQA12 | <--- | F1 | .617 |
| EDAQA22 | <--- | F1 | .668 |
| EDAQA21 | <--- | F1 | .695 |
| EDAQA2 | <--- | F1 | .655 |
| EDAQA4 | <--- | F1 | .655 |
| EDAQA13 | <--- | F1 | .647 |
| EDAQA9 | <--- | F1 | .648 |
| EDAQA19 | <--- | F1 | .696 |
| EDAQA18 | <--- | F1 | .652 |
| EDAQA26 | <--- | F1 | .599 |
| EDAQA6 | <--- | F1 | .623 |
| EDAQA8 | <--- | F2 | .646 |
| EDAQA10 | <--- | F2 | .590 |
| EDAQA24 | <--- | F2 | .669 |

**Covariances: (Group number 1 - Default model)**

|  |  |  | Estimate | S.E. | C.R. | P | Label |
| --- | --- | --- | --- | --- | --- | --- | --- |
| ef1 | <--> | ef2 | .307 | .038 | 8.181 | *** |  |
| e1 | <--> | e10 | .223 | .039 | 5.658 | *** |  |
| e15 | <--> | e16 | .222 | .042 | 5.317 | *** |  |
| e14 | <--> | e20 | .147 | .036 | 4.042 | *** |  |
| e8 | <--> | e16 | .203 | .038 | 5.358 | *** |  |
| e8 | <--> | e15 | .216 | .038 | 5.653 | *** |  |
| e3 | <--> | e13 | .165 | .036 | 4.622 | *** |  |
| e1 | <--> | e21 | -.146 | .039 | -3.710 | *** |  |
| e1 | <--> | e4 | .138 | .036 | 3.810 | *** |  |
| e23 | <--> | e24 | .237 | .055 | 4.350 | *** |  |
| e14 | <--> | e18 | .127 | .036 | 3.504 | *** |  |
| e6 | <--> | e16 | .149 | .036 | 4.170 | *** |  |
| e6 | <--> | e8 | .101 | .031 | 3.213 | .001 |  |
| e9 | <--> | e19 | .070 | .032 | 2.199 | .028 |  |
| e5 | <--> | e19 | -.092 | .036 | -2.578 | .010 |  |
| e13 | <--> | e17 | .178 | .041 | 4.296 | *** |  |
| e13 | <--> | e16 | .104 | .033 | 3.102 | .002 |  |
| e5 | <--> | e10 | .128 | .041 | 3.098 | .002 |  |
| e1 | <--> | e7 | .115 | .033 | 3.455 | *** |  |
| e17 | <--> | e18 | .128 | .041 | 3.152 | .002 |  |
| e9 | <--> | e17 | -.089 | .033 | -2.658 | .008 |  |
| e3 | <--> | e17 | .116 | .037 | 3.157 | .002 |  |
| e2 | <--> | e22 | -.139 | .041 | -3.355 | *** |  |
| e1 | <--> | e14 | .108 | .031 | 3.525 | *** |  |
| e19 | <--> | e21 | .148 | .042 | 3.540 | *** |  |
| e9 | <--> | e20 | -.115 | .034 | -3.404 | *** |  |
| e20 | <--> | e21 | .157 | .046 | 3.446 | *** |  |

**Correlations: (Group number 1 - Default model)**

|  |  |  | Estimate |
| --- | --- | --- | --- |
| ef1 | <--> | ef2 | .636 |
| e1 | <--> | e10 | .304 |
| e15 | <--> | e16 | .302 |
| e14 | <--> | e20 | .222 |
| e8 | <--> | e16 | .317 |
| e8 | <--> | e15 | .336 |
| e3 | <--> | e13 | .275 |
| e1 | <--> | e21 | -.177 |
| e1 | <--> | e4 | .198 |
| e23 | <--> | e24 | .346 |
| e14 | <--> | e18 | .189 |
| e6 | <--> | e16 | .229 |
| e6 | <--> | e8 | .176 |
| e9 | <--> | e19 | .125 |
| e5 | <--> | e19 | -.139 |
| e13 | <--> | e17 | .246 |
| e13 | <--> | e16 | .146 |
| e5 | <--> | e10 | .168 |
| e1 | <--> | e7 | .179 |
| e17 | <--> | e18 | .168 |
| e9 | <--> | e17 | -.139 |
| e3 | <--> | e17 | .183 |
| e2 | <--> | e22 | -.193 |
| e1 | <--> | e14 | .170 |
| e19 | <--> | e21 | .195 |
| e9 | <--> | e20 | -.182 |
| e20 | <--> | e21 | .183 |

**Variances: (Group number 1 - Default model)**

|  |  |  | Estimate | S.E. | C.R. | P | Label |
| --- | --- | --- | --- | --- | --- | --- | --- |
| ef1 |  |  | .552 | .045 | 12.258 | *** |  |
| ef2 |  |  | .423 | .054 | 7.841 | *** |  |
| e1 |  |  | .687 | .054 | 12.708 | *** |  |
| e2 |  |  | .601 | .049 | 12.294 | *** |  |
| e3 |  |  | .528 | .043 | 12.194 | *** |  |
| e4 |  |  | .704 | .056 | 12.463 | *** |  |
| e5 |  |  | .743 | .060 | 12.466 | *** |  |
| e6 |  |  | .580 | .047 | 12.300 | *** |  |
| e7 |  |  | .600 | .049 | 12.343 | *** |  |
| e8 |  |  | .561 | .046 | 12.323 | *** |  |
| e9 |  |  | .530 | .044 | 12.148 | *** |  |
| e10 |  |  | .786 | .062 | 12.575 | *** |  |
| e12 |  |  | .898 | .071 | 12.617 | *** |  |
| e13 |  |  | .684 | .055 | 12.455 | *** |  |
| e14 |  |  | .589 | .048 | 12.388 | *** |  |
| e15 |  |  | .733 | .059 | 12.480 | *** |  |
| e16 |  |  | .734 | .058 | 12.617 | *** |  |
| e17 |  |  | .767 | .062 | 12.465 | *** |  |
| e18 |  |  | .763 | .061 | 12.523 | *** |  |
| e19 |  |  | .588 | .048 | 12.227 | *** |  |
| e20 |  |  | .747 | .060 | 12.482 | *** |  |
| e21 |  |  | .987 | .078 | 12.690 | *** |  |
| e22 |  |  | .869 | .069 | 12.547 | *** |  |
| e23 |  |  | .592 | .061 | 9.687 | *** |  |
| e24 |  |  | .794 | .076 | 10.459 | *** |  |
| e25 |  |  | .523 | .055 | 9.530 | *** |  |

**Modification Indices (Group number 1 - Default model)**

**Covariances: (Group number 1 - Default model)**

|  |  |  | M.I. | Par Change |
| --- | --- | --- | --- | --- |
| e22 | <--> | ef2 | 19.790 | .161 |
| e22 | <--> | ef1 | 11.409 | -.115 |
| e22 | <--> | e23 | 27.524 | .205 |
| e20 | <--> | ef2 | 5.351 | .073 |
| e20 | <--> | ef1 | 4.807 | -.065 |
| e19 | <--> | ef2 | 4.468 | .061 |
| e19 | <--> | ef1 | 17.245 | -.113 |
| e18 | <--> | ef1 | 9.049 | -.092 |
| e17 | <--> | ef2 | 4.076 | .064 |
| e16 | <--> | e19 | 4.607 | .067 |
| e14 | <--> | e17 | 5.623 | .077 |
| e14 | <--> | e15 | 7.243 | .083 |
| e13 | <--> | e20 | 4.515 | -.073 |
| e13 | <--> | e15 | 5.497 | .077 |
| e12 | <--> | e18 | 8.060 | .126 |
| e9 | <--> | e25 | 7.863 | .092 |
| e9 | <--> | e14 | 4.670 | -.062 |
| e7 | <--> | e20 | 5.880 | .084 |
| e7 | <--> | e19 | 4.186 | -.065 |
| e7 | <--> | e17 | 6.593 | -.089 |
| e6 | <--> | ef2 | 8.074 | -.081 |
| e6 | <--> | ef1 | 8.871 | .080 |
| e6 | <--> | e25 | 12.537 | -.118 |
| e6 | <--> | e20 | 5.427 | -.078 |
| e6 | <--> | e12 | 4.911 | .086 |
| e5 | <--> | e22 | 9.092 | -.132 |
| e4 | <--> | ef2 | 6.105 | -.079 |
| e4 | <--> | e23 | 13.882 | -.129 |
| e3 | <--> | ef2 | 8.639 | -.079 |
| e3 | <--> | ef1 | 8.550 | .074 |
| e3 | <--> | e25 | 4.928 | -.070 |
| e3 | <--> | e23 | 4.039 | -.059 |
| e3 | <--> | e10 | 4.178 | .064 |
| e2 | <--> | ef1 | 10.599 | .093 |
| e2 | <--> | e12 | 5.506 | .097 |
| e2 | <--> | e6 | 7.751 | .089 |
| e1 | <--> | e5 | 4.174 | .069 |

**Variances: (Group number 1 - Default model)**

|  |  |  | M.I. | Par Change |
| --- | --- | --- | --- | --- |

**Regression Weights: (Group number 1 - Default model)**

|  |  |  | M.I. | Par Change |
| --- | --- | --- | --- | --- |
| EDAQA24 | <--- | EDAQA23 | 6.492 | .109 |
| EDAQA8 | <--- | EDAQA6 | 16.931 | .143 |
| EDAQA8 | <--- | EDAQA3 | 8.518 | -.108 |
| EDAQA6 | <--- | F2 | 4.031 | .187 |
| EDAQA6 | <--- | EDAQA8 | 22.476 | .239 |
| EDAQA6 | <--- | EDAQA12 | 4.351 | -.088 |
| EDAQA6 | <--- | EDAQA17 | 8.553 | -.131 |
| EDAQA26 | <--- | F2 | 4.788 | -.204 |
| EDAQA26 | <--- | EDAQA10 | 4.484 | -.097 |
| EDAQA26 | <--- | EDAQA8 | 6.570 | -.129 |
| EDAQA26 | <--- | EDAQA2 | 5.215 | -.102 |
| EDAQA26 | <--- | EDAQA12 | 4.801 | -.092 |
| EDAQA26 | <--- | EDAQA17 | 4.107 | -.090 |
| EDAQA26 | <--- | EDAQA15 | 4.910 | -.108 |
| EDAQA18 | <--- | EDAQA10 | 5.760 | .097 |
| EDAQA18 | <--- | EDAQA8 | 4.712 | .096 |
| EDAQA18 | <--- | EDAQA22 | 5.018 | -.090 |
| EDAQA18 | <--- | EDAQA7 | 6.465 | -.107 |
| EDAQA19 | <--- | F1 | 12.187 | -.197 |
| EDAQA19 | <--- | EDAQA26 | 4.335 | -.068 |
| EDAQA19 | <--- | EDAQA18 | 9.589 | -.110 |
| EDAQA19 | <--- | EDAQA9 | 13.023 | -.128 |
| EDAQA19 | <--- | EDAQA2 | 4.643 | -.077 |
| EDAQA19 | <--- | EDAQA21 | 12.632 | -.135 |
| EDAQA19 | <--- | EDAQA12 | 6.691 | -.087 |
| EDAQA19 | <--- | EDAQA16 | 5.574 | -.083 |
| EDAQA19 | <--- | EDAQA23 | 6.161 | -.097 |
| EDAQA19 | <--- | EDAQA1 | 6.892 | -.101 |
| EDAQA19 | <--- | EDAQA5 | 15.083 | -.147 |
| EDAQA19 | <--- | EDAQA17 | 4.905 | -.079 |
| EDAQA19 | <--- | EDAQA3 | 14.386 | -.137 |
| EDAQA19 | <--- | EDAQA25 | 11.345 | -.127 |
| EDAQA19 | <--- | EDAQA11 | 11.477 | -.123 |
| EDAQA9 | <--- | F2 | 8.087 | -.239 |
| EDAQA9 | <--- | F1 | 13.098 | -.231 |
| EDAQA9 | <--- | EDAQA24 | 4.306 | -.098 |
| EDAQA9 | <--- | EDAQA6 | 7.302 | -.104 |
| EDAQA9 | <--- | EDAQA26 | 7.340 | -.100 |
| EDAQA9 | <--- | EDAQA19 | 16.086 | -.172 |
| EDAQA9 | <--- | EDAQA13 | 4.836 | -.088 |
| EDAQA9 | <--- | EDAQA4 | 11.644 | -.138 |
| EDAQA9 | <--- | EDAQA2 | 12.333 | -.142 |
| EDAQA9 | <--- | EDAQA21 | 5.559 | -.101 |
| EDAQA9 | <--- | EDAQA22 | 8.887 | -.123 |
| EDAQA9 | <--- | EDAQA23 | 11.676 | -.151 |
| EDAQA9 | <--- | EDAQA1 | 8.373 | -.126 |
| EDAQA9 | <--- | EDAQA7 | 11.335 | -.145 |
| EDAQA9 | <--- | EDAQA17 | 4.099 | -.082 |
| EDAQA9 | <--- | EDAQA3 | 4.901 | -.091 |
| EDAQA9 | <--- | EDAQA25 | 9.458 | -.131 |
| EDAQA13 | <--- | EDAQA24 | 4.076 | .093 |
| EDAQA13 | <--- | EDAQA21 | 5.165 | .095 |
| EDAQA4 | <--- | EDAQA12 | 4.878 | -.076 |
| EDAQA4 | <--- | EDAQA16 | 6.433 | -.090 |
| EDAQA4 | <--- | EDAQA3 | 4.678 | -.079 |
| EDAQA4 | <--- | EDAQA25 | 5.218 | -.088 |
| EDAQA4 | <--- | EDAQA11 | 4.084 | -.075 |
| EDAQA21 | <--- | EDAQA23 | 6.495 | -.096 |
| EDAQA22 | <--- | EDAQA8 | 4.200 | .084 |
| EDAQA22 | <--- | EDAQA2 | 5.064 | .082 |
| EDAQA22 | <--- | EDAQA23 | 4.789 | .087 |
| EDAQA12 | <--- | EDAQA9 | 5.238 | .104 |
| EDAQA12 | <--- | EDAQA25 | 4.010 | .097 |
| EDAQA23 | <--- | EDAQA24 | 7.676 | .112 |
| EDAQA23 | <--- | EDAQA4 | 5.891 | .084 |
| EDAQA23 | <--- | EDAQA22 | 4.573 | .076 |
| EDAQA23 | <--- | EDAQA3 | 5.218 | .080 |
| EDAQA23 | <--- | EDAQA25 | 6.657 | .095 |
| EDAQA1 | <--- | EDAQA25 | 4.454 | .072 |
| EDAQA5 | <--- | EDAQA18 | 8.507 | .107 |
| EDAQA5 | <--- | EDAQA3 | 4.865 | .082 |
| EDAQA5 | <--- | EDAQA25 | 4.744 | .085 |
| EDAQA7 | <--- | EDAQA24 | 6.885 | -.108 |
| EDAQA7 | <--- | EDAQA22 | 7.978 | .102 |
| EDAQA7 | <--- | EDAQA12 | 7.445 | .091 |
| EDAQA7 | <--- | EDAQA23 | 4.415 | .081 |
| EDAQA7 | <--- | EDAQA3 | 4.144 | .073 |
| EDAQA7 | <--- | EDAQA15 | 7.770 | .108 |
| EDAQA7 | <--- | EDAQA25 | 9.120 | .113 |
| EDAQA17 | <--- | EDAQA6 | 4.076 | -.078 |
| EDAQA17 | <--- | EDAQA11 | 6.154 | .103 |
| EDAQA3 | <--- | EDAQA8 | 12.019 | -.155 |
| EDAQA15 | <--- | EDAQA16 | 9.153 | .099 |
| EDAQA15 | <--- | EDAQA1 | 4.235 | .074 |
| EDAQA15 | <--- | EDAQA7 | 6.202 | .089 |
| EDAQA15 | <--- | EDAQA11 | 4.967 | .076 |
| EDAQA25 | <--- | F1 | 9.978 | .188 |
| EDAQA25 | <--- | EDAQA12 | 13.703 | .131 |
| EDAQA25 | <--- | EDAQA16 | 6.557 | .095 |
| EDAQA25 | <--- | EDAQA23 | 12.695 | .146 |
| EDAQA25 | <--- | EDAQA1 | 8.030 | .115 |
| EDAQA25 | <--- | EDAQA5 | 11.736 | .136 |
| EDAQA25 | <--- | EDAQA7 | 16.803 | .165 |
| EDAQA25 | <--- | EDAQA17 | 7.370 | .102 |
| EDAQA25 | <--- | EDAQA3 | 11.267 | .128 |
| EDAQA25 | <--- | EDAQA11 | 11.235 | .129 |

**Minimization History (Default model)**

| Iteration |  | Negative eigenvalues | Condition # | Smallest eigenvalue | Diameter | F | NTries | Ratio |
| --- | --- | --- | --- | --- | --- | --- | --- | --- |
| 0 | e | 21 |  | -.278 | 9999.000 | 2341.205 | 0 | 9999.000 |
| 1 | e* | 1 |  | -.079 | 1.485 | 985.324 | 18 | .951 |
| 2 | e | 0 | 104.269 |  | .465 | 701.140 | 5 | .864 |
| 3 | e | 0 | 29.404 |  | .780 | 542.077 | 2 | .000 |
| 4 | e | 0 | 24.428 |  | .150 | 529.246 | 1 | 1.070 |
| 5 | e | 0 | 23.020 |  | .041 | 528.555 | 1 | 1.062 |
| 6 | e | 0 | 23.277 |  | .005 | 528.548 | 1 | 1.008 |
| 7 | e | 0 | 23.264 |  | .000 | 528.548 | 1 | 1.000 |

**Model Fit Summary**

**CMIN**

| Model | NPAR | CMIN | DF | P | CMIN/DF |
| --- | --- | --- | --- | --- | --- |
| Default model | 53 | 528.548 | 247 | .000 | 2.140 |
| Saturated model | 300 | .000 | 0 |  |  |
| Independence model | 24 | 4534.020 | 276 | .000 | 16.428 |

**RMR, GFI**

| Model | RMR | GFI | AGFI | PGFI |
| --- | --- | --- | --- | --- |
| Default model | .098 | .888 | .864 | .731 |
| Saturated model | .000 | 1.000 |  |  |
| Independence model | .489 | .200 | .131 | .184 |

**Baseline Comparisons**

| Model | NFI Delta1 | RFI rho1 | IFI Delta2 | TLI rho2 | CFI |
| --- | --- | --- | --- | --- | --- |
| Default model | .883 | .870 | .934 | .926 | .934 |
| Saturated model | 1.000 |  | 1.000 |  | 1.000 |
| Independence model | .000 | .000 | .000 | .000 | .000 |

**Parsimony-Adjusted Measures**

| Model | PRATIO | PNFI | PCFI |
| --- | --- | --- | --- |
| Default model | .895 | .791 | .836 |
| Saturated model | .000 | .000 | .000 |
| Independence model | 1.000 | .000 | .000 |

**NCP**

| Model | NCP | LO 90 | HI 90 |
| --- | --- | --- | --- |
| Default model | 281.548 | 219.177 | 351.667 |
| Saturated model | .000 | .000 | .000 |
| Independence model | 4258.020 | 4043.453 | 4479.860 |

**FMIN**

| Model | FMIN | F0 | LO 90 | HI 90 |
| --- | --- | --- | --- | --- |
| Default model | 1.528 | .814 | .633 | 1.016 |
| Saturated model | .000 | .000 | .000 | .000 |
| Independence model | 13.104 | 12.306 | 11.686 | 12.948 |

**RMSEA**

| Model | RMSEA | LO 90 | HI 90 | PCLOSE |
| --- | --- | --- | --- | --- |
| Default model | .057 | .051 | .064 | .036 |
| Independence model | .211 | .206 | .217 | .000 |

**AIC**

| Model | AIC | BCC | BIC | CAIC |
| --- | --- | --- | --- | --- |
| Default model | 634.548 | 642.803 | 838.562 | 891.562 |
| Saturated model | 600.000 | 646.729 | 1754.797 | 2054.797 |
| Independence model | 4582.020 | 4585.758 | 4674.404 | 4698.404 |

**ECVI**

| Model | ECVI | LO 90 | HI 90 | MECVI |
| --- | --- | --- | --- | --- |
| Default model | 1.834 | 1.654 | 2.037 | 1.858 |
| Saturated model | 1.734 | 1.734 | 1.734 | 1.869 |
| Independence model | 13.243 | 12.623 | 13.884 | 13.254 |

**HOELTER**

| Model | HOELTER .05 | HOELTER .01 |
| --- | --- | --- |
| Default model | 187 | 198 |
| Independence model | 25 | 26 |

**Execution time summary**

| Minimization: | .017 |
| --- | --- |
| Miscellaneous: | 1.486 |
| Bootstrap: | .000 |
| Total: | 1.503 |
